# Synthesis, Properties and Bioimaging Applications of Silver-Based Quantum Dots

**DOI:** 10.3390/ijms222212202

**Published:** 2021-11-11

**Authors:** Mariya Borovaya, Inna Horiunova, Svitlana Plokhovska, Nadia Pushkarova, Yaroslav Blume, Alla Yemets

**Affiliations:** Institute of Food Biotechnology and Genomics, National Academy of Sciences of Ukraine, Osypovskoho Str. 2a, 04123 Kyiv, Ukraine; inna.horiunova.ukr@gmail.com (I.H.); svetaplohovska@gmail.com (S.P.); pushkarovano@gmail.com (N.P.); blume.yaroslav@nas.gov.ua (Y.B.); yemets.alla@nas.gov.ua (A.Y.)

**Keywords:** silver-based QDs, photoluminescence, near-infrared, “green” synthesis, fluorescence imaging

## Abstract

Ag-based quantum dots (QDs) are semiconductor nanomaterials with exclusive electrooptical properties ideally adaptable for various biotechnological, chemical, and medical applications. Silver-based semiconductor nanocrystals have developed rapidly over the past decades. They have become a promising luminescent functional material for in vivo and in vitro fluorescent studies due to their ability to emit at the near-infrared (NIR) wavelength. In this review, we discuss the basic features of Ag-based QDs, the current status of classic (chemical) and novel methods (“green” synthesis) used to produce these QDs. Additionally, the advantages of using such organisms as bacteria, actinomycetes, fungi, algae, and plants for silver-based QDs biosynthesis have been discussed. The application of silver-based QDs as fluorophores for bioimaging application due to their fluorescence intensity, high quantum yield, fluorescent stability, and resistance to photobleaching has also been reviewed.

## 1. Introduction

Nanometer-sized semiconductor crystals or ‘quantum dots’ (QDs) have been extensively studied. They have become an extremely successful nanoscale material for a wide range of applications such as fluorescent essays or disease detection due to their unique fluorescent electronic characteristics, mainly simultaneous excitation in multiple spectra of colors, size-dependent light radiation, long-term photostability, and high signal brightness [1]. As the particle size grows, part of the surface atoms also gets larger. Consequently, the continuum of the electronic state becomes discrete (the so-called “quantum size effect”), which leads to the loss of a significant part of the previous properties [2]. Regardless of the size of the QDs and their chemical composition, a wide range of the light spectrum (from visible to infrared) of different wavelengths is emitted. A group of Cd-, As-, Hg- and Pb-containing QDs (II–VI or III–V QDs) are not suitable for biological application due to their high toxicity caused by heavy metal ions and reactive oxygen species (ROS) they contained. To eliminate this obstacle, Cd-free QDs are recommended, which have preferable properties and can synthesize tunable emission spectra [2]. Silver-based semiconductor QDs attract much attention due to their suitability for many practical biomedical applications, such as drug or gene delivery, high-precision diagnostics, microscopic studies, etc. [3]. Moreover, nanostructured silver-containing QD composites are applied to develop systems for identifying and quantifying different pathogenic microorganisms, toxic chemicals, or molecular species in small concentrations using surface-enhanced Raman spectroscopy (SERS) [4,5,6,7,8,9,10].

Among the silver-containing QDs—Ag_2_S, Ag_2_Se, Ag_2_Te and ZnAgInSe, AgInS_2_, AgInSe_2_—nanocrystals are usually differentiated. There are two approaches to QDs synthesis: the bottom-up approach starts at a molecular level, has a high molecular structure control, and is suitable for forming larger and complex systems, and a top-down approach in which larger QDs are used to assemble the smaller ones [3]. For silver-based QDs biosynthesis, the bottom-up approach is used, the main reactions of which are reduction/oxidation. The reduction of a metal compound into its respective nanomaterial is performed through the plant, fungal or microbial matrices that possess antioxidative or reducing properties. We have previously developed successful and easily reproducible methods of extracellular “green” synthesis of CdS and Ag_2_S QDs based on fungal and plant matrices [11,12,13,14,15]. “Green” synthesis of silver-containing QDs using no toxic chemical compounds makes the obtained nanomaterials suitable for biological and biomedical research. Not least importantly, they are low cost and environmentally friendly [16,17]. Besides, Ag-containing QDs are frequently used for bioimaging examinations. They possess all the ideal features of fluorescent agents used in biomedicine—the ability to emit fluorescence with high quantum yield (QY) and high stability cytocompatibility. Traditional fluorescent probes, applied in biomedicine, use rhodamines, fluoresceins, or cyanines as dyes despite some restrictions (sensitivity to photobleaching, low photochemical stability, short lifespan of fluorescence). In contrast, QDs are characterized with advantageous physical, chemical, and optical properties due to their particle size, unique optical qualities, and quantum duress [18]. This review aims to bring together the currently available research data on the physical properties, novel “green” synthesis techniques of silver-based QDs mediated by bacteria, fungi, plants, different biomolecules, and also to highlight the wide application range of Ag-based QDs in biomedicine for in vivo and in vitro cellular labeling.

## 2. Basic Properties of Ag-Based QDs

Specific physicochemical properties of QDs are usually associated with the quantum confinement phenomenon [19]. Quantum confinement is manifested by changing the energy of the electronic spectrum of materials under the transition to the nanoscale level. The most important feature of QDs is the dependence of their optical spectrum on the size—when the physical size of the material decreases to nanoscales, the electronic excitation shifts to a higher energy level, thereby making the energy spectrum discrete. The optical features of QDs include a high level of photons absorption, stable prolonged photoluminescence (PL), tunneling, and luminescence emission with a large Stokes shift [20]. It should be noted that non-cadmium-based QDs are NIR emitting, which is preferable for living tissues imaging due to the deep light penetration and the decay autofluorescence in the infrared region at about 750–940 nm (NIR-I window) and 1000–1700 nm (NIR II window) wavelengths.

**Figure 1:**
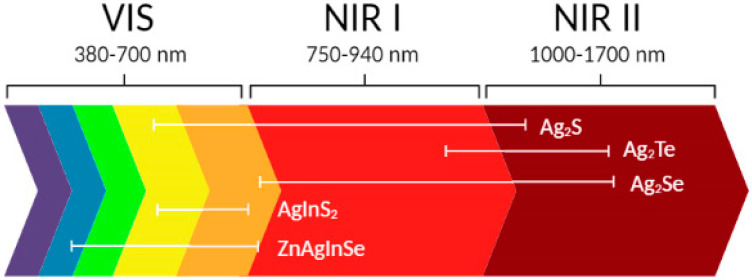
Emission wavelength range of Ag-based QDs.

Ag_2_S have emerged as novel NIR-II emitting QDs with preferable photo- and thermoelectric features, great catalytic properties, and electrical conduction having almost no toxicity and a band gap of ~1 eV [21]. Nanoscale Ag_2_S is a biocompatible semiconductor material with an asymmetric emission spectrum of light characterized by high stability and intensity and exhibits high magnetoresistance. The light emitted in the 700–900 nm region is considered an optical imaging window with a high depth of light penetration into the tissue and a minimal absorption [22,23] (Figure 1).

Ag_2_S QDs are distinguished by different crystal structures and are classified into the following types:

- Monoclinic α-Ag_2_S QDs (body-centered cubic, stable at 178 °C and below);
- β-Ag_2_S (face-centered cubic, stable at 178–600 °C);
- γ-Ag_2_S (stable at 600 °C and above).

For this reason, Ag_2_S has become highly popular in nanomedicine due to its stability, strong luminescence, and high compatibility with biological samples [24]. Silver chalcogenides have recently been suggested as NIR-II emitting QDs due to their highly restricted band gaps (0.9 eV for Ag_2_S, 0.15 eV for Ag_2_Se, and 0.67 eV for Ag_2_Te) and low toxicity [25,26]. Out of these NPs, Ag_2_Se is preferable due to its low toxicity compared to other NIR-II QDs (HgS, HgSe, PbS, heterostructure QDs). Due to the potential possibility to combine NIR and magnetic resonance imaging, the promising potential of NIR-II photoluminescence of Ag_2_Se QDs for dual imaging is increasing.

Small water-soluble NIR-I and NIR-II Ag_2_Se, as well as non-fluorescent Ag_2_Se QDs were synthesized earlier. Still, the issue of tuning their emission in the second near-infrared window for possible use in polychrome labeling for simultaneous imaging of multiple targets exists [27]. Ag_2_Se QDs obtained at different reaction times varied greatly in size—3.1 nm, 3.4 nm, and 3.9 nm particle diameter at the reaction time of 1 min, 5 min, and 1 h, respectively. That was established by transmission electron microscopy (TEM) while the powder X-ray diffraction (XRD) pattern showed that QDs diffraction peaks coincided with orthorhombic Ag_2_Se. Wide peaks could be associated with the small particles’ crystallinity. Optical features of Ag_2_Se QDs were examined using UV-vis-NIR and fluorescence spectrophotometry. With an increased reaction time, Ag_2_Se QDs exhibited red-shift absorption (in 770–1070 nm) and red-shift fluorescence emission (in 1080–1330 nm) [28,29]. β-Ag_2_Se could be easily tuned to the NIR-II region by changing its size. With no heavy metal component and optimal emission peak at 1300 nm, β-Ag_2_Se could be well suited for in vivo imaging. Given its small size, suitable optical characteristics, and higher biocompatibility, this NIR-II fluorescent QD opens up great opportunities in biomedicine [28].

Due to their restricted band gaps of ~0.67 eV, Ag_2_Te QDs are considered a great source of low toxicity NIR emissive nanocrystals. Despite the fact that Ag_2_Te QDs belong to the silver chalcogenide group, their properties are less studied than Ag_2_S and Ag_2_Se [30]. Metal tellurides are of great interest due to their exceptional characteristics and utilization in such cases as magnetic-field measurements, structural studies, and microelectronics [31,32]. Ag_2_Te demonstrates unique optical properties, such as optical filters based on Ag_2_Te thin films, increases the efficiency of Raman scattering in β-Ag_2_Te nanotubes, and studies of polarized Raman spectroscopy in β-Ag_2_Te phase [33]. Ag_2_Te also possesses high magnetoresistance. It was previously stated that Ag_2_Te nanomaterials if prepared in organic solvents and treated by ligand exchange and surface rearrangement, should obtain hydrophilic properties and thus could be suitable for bio-application [34]. For example, Ag_2_Te nanowires with a single crystalline nature are valuable for higher electrical conductivity, which is considered an excellent feature for thermoelectrical application [35]. Several Ag_2_Te nano-wires/tubes/rods/particles were brought to light. However, almost no emphasis was made on luminescent Ag_2_Te QDs, and insufficient efforts were made to develop them as a tool for imaging in the NIR-II region [34,36]. These QDs are characterized by thermoelectric features (high mobility of electrons and low thermal conductivity) [37]. The compact hydrodynamic dimensions of Ag_2_Te QDs provide it with high QYs and colloidal/photostability. These QDs are shown to be highly biocompatible due to extremely low cytotoxicity and could be used for top-quality NIR-II PL imaging in cells [30].

Ternary metal dichalcogenides QDs (CuInS_2_ and AgInS_2_) are currently of interest as an alternative to binary cadmium-containing and lead-containing chalcogenide QDs, and due to their low toxicity, are also considered to be environmentally safe and are extensively used in biocompatible devices [38,39,40]. Ternary and quaternary silver-based QDs recently gained recognition due to their superior photophysical properties [41]. The main features of ternary QDs are prolonged PL lifespan, huge Stokes shifts, and their resistance to defect states, leading to a broadband absorption formation and emission. It should be noted that optical features of AgInS_2_ QDs depend on their size owing to the quantum confinement effect. For previously synthesized in aqueous and organic media AgInS_2_/Zn, PL excitation was shown at 405 nm with its maximum at 562 nm, 532 nm for hydrophilic, and 560 and 550 nm for hydrophobic QDs [42].

Great interest is focused on the Ag-B^III^-C^VI^ ternary group [B^III^-Al, In, Ga; C^VI^-S, Se, Te] family compounds due to their characteristics promising for applying in photoelectric devices—high radiation stability, considerable extinction coefficient (up to 105 cm^−1^), and the direct band gap. AgInSe_2_ is considered a potential tool as a photosensitizer for QDs-sensitive solar cells because its compounds are less toxic than heavy metal cations such as Pb^2+^, Cd^2+^ or Hg^2+,^ and it has a band gap of 1.2 eV [43]. Despite its promising application, only a few reports on these ternary QDs preparations exist. AgInSe_2_ QDs synthesis with fluorescence spectrum from orange to red and PL peak < 700 nm [44]; with broad trap emission 950–1250 nm (the second NIR window) which were obtained based on the thermolysis of an Ag-In-thiolate complex with a subsequent anion exchange [45].

**Figure 2:**
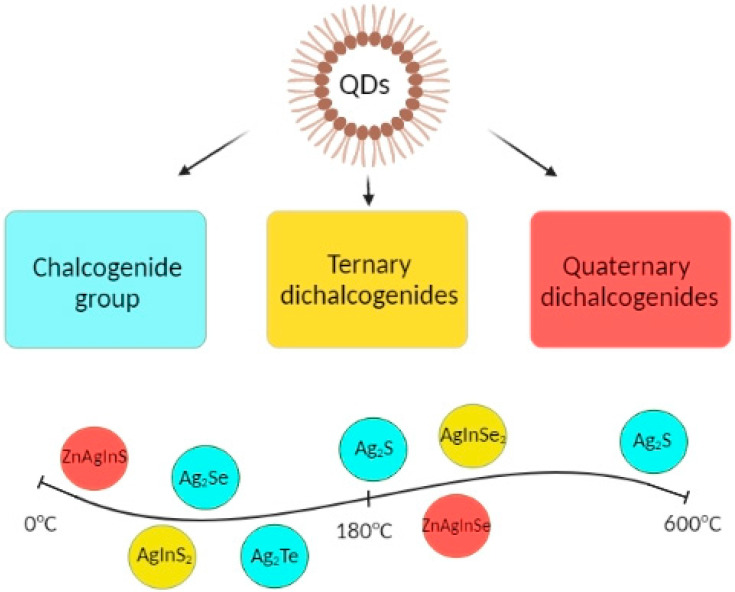
Schematic diagram of main types of Ag-based QDs.

Another promising QD type are quaternary ones, specifically ZnAgInSe QDs [46]. Freshly made quaternary QDs show composition-tunable bright PL with wide spectrum, specifically, QY up to 30% and PL peak was measured at 450–760 nm [47]. Classification of Ag-based QDs is presented in Table 1 and Figure 2.

Analyzing the properties of existing Ag-containing quantum dots, we can conclude that most of them are NIR emitted (this optical imaging region possesses a high depth of light penetration into the tissue with minimal autofluorescence), less toxic, heavy metals free, have prolonged luminescence. Depending on the synthesis conditions, silver-based nanoparticles have different morphology sizes and properties [49]. Considering their basic optical features, these QDs have great potential for use in optoelectronic devices and biomedical research.

## 3. Chemical Synthesis of Ag-Based QDs

The search and development of new possible directions for QDs synthesis is important for nanotechnology progress. Methods for the synthesis of organometallic and other organic solutions for the preparation of cadmium-containing QDs were described in [3]. This part of the article summarizes several chemical methods for synthesizing Ag-based quantum dots, such as the single-source precursor technique, hydro-chemical deposition, microwave irradiation, sol-gel method, embedded particles technology template method, etc. As described by Zhao et al., Ag_2_S nanocrystalline were synthetized from Na_2_S_2_O_3_ as a chalcogen source by irradiation at room temperature. Polyvinylpyrrolidone (PVP) is fairly important for the rod-like Ag_2_S nanocrystalline and has a role in crystal growth guiding reagent [50].

The leaf-like Ag_2_S nanosheets were prepared by Chen et al. using a facile hydrothermal method in an alcohol-water homogenous medium. The facile one-step method applied at room temperature allowed the production of various morphologies of Ag_2_S micro- and nanomaterials (micrometer bars, nanowires, nanopolyhedrons) without the addition of organic template materials to the reaction container. To obtain QDs of different sizes and morphology, the Ag^+^, S^2−^ and ammonia ratio was alternated [51]. Chaudhuri et al. proposed another simple method for synthesizing hollow Ag_2_S particles using a sacrificial core method in aqueous media containing surfactant [52].

The method of synthesis of the one-molecular precursor of nanomaterials based on Ag has some advantages—it is simple, safe, and demonstrates specific compatibility with the metalorganic chemical vapor deposition. However, the molecular precursor could correspond to the unusual selectivity of crystal growth and metastable phase formation of the received products, which are difficult to obtain using synthesis methods [21].

Another approach is the sol-gel method which has received a certain recognition and a wide practical application for advanced materials production, including oxide-based coatings [53]. Hydrolysis and condensation reactions lead to the formation of oxo-based macromolecular networks that allow the selection of precursor compounds in accordance with the necessary process. This method is also characterized by mild conditions of synthesis—so-called “soft chemistry”. Therefore, the sol-gel method is particularly suitable for the production of thin films with good microstructure and compound control [54,55].

The use of microwave energy to heat chemical reactions is a widely accepted method, especially in the field of material science, nanotechnology, biochemical processes, etc. For instance, Ag_2_S-AgInS_2_ nanocomposites were prepared from a solution of propylene glycol using microwave energy [56]. Ag_2_Se nanocrystals are most commonly synthesized using co-precipitation. This effective method involves solutions of the original precursors in combination with the subsequent addition of a precipitator to precipitate Ag_2_Se QDs. As shown by TEM, the fluorescence of these QDs’ was observed in the NIR-II window (1080–1330 nm), and the size distribution of nanocrystals ranged from 5 to 31.5 nm [28].

The hydrothermal method is usually applied to obtain a variety of Ag_2_Te nanostructures using hydrazine monohydrate (reducing agent) and TeCl_4_ (precursor) [57]. The solvothermal method as a simple surfactant-assisted way for the synthesis of CoTe, Ag_2_Te/Ag, and CdTe QDs synthesis is conducted using corresponding metal salt and reported in detail in [30].

The hydrothermal method of the microwave oven is an efficient and fast way to obtain AgInS_2_ NPs without the use of organic solvents, catalysts, and surfactants. The formed QDs are characterized by the fine photocatalytic activity of visible light and have a particle diameter of 20–80 nm. AgInS_2_ can be in two crystalline forms—orthorhombic (stable at temperatures above 620 °C) and tetragonal or chalcopyrite (stable at temperatures below 620 °C). This method of NPs synthesis allows the production of metastable orthorhombic AgInS_2_ QDs even at 200 °C and below. Therefore, metastable phases which are excluded by conventional methods could be synthesized using microwave irradiation [58]. The water-soluble ZnAgInSe NPs can be prepared in an aqueous phase in the absence of highly toxic heavy metal elements and with the addition of glutathione as a stabilizing agent. The as-prepared quaternary QDs exhibit a widely tunable composition and bright PL [45].

On the whole, despite some limitations and disadvantages, for example, high temperature, complicated processes, gas atmosphere, and difficulty in controlling the desired particle size, the chemical preparation process of Ag-based QDs is widespread. Recently, substantial work and effort have been made to optimize the synthesis process of Ag_2_S and other Ag-based NPs to enhance their properties and values of products. Chemical synthesis methods, PL, and morphological features of Ag-based QDs are summarized below in Table 2.

## 4. “Green” Synthesis of Ag-Based QDs

“Green” synthesis using natural resources, plant materials, or parts of plants and their extracts, cultures of microorganisms, or fungi have proven to be an effective biological matrix usually used for the preparation of QDs of different chemical compositions. In addition, synthesis using biological matrices or biocompounds is cheaper since it does not require specific chemical solvents and stabilizers. In general, “green” synthesis is more efficient because its application leads to obtaining environmentally friendly high-quality nanomaterials.

**Figure 3:**
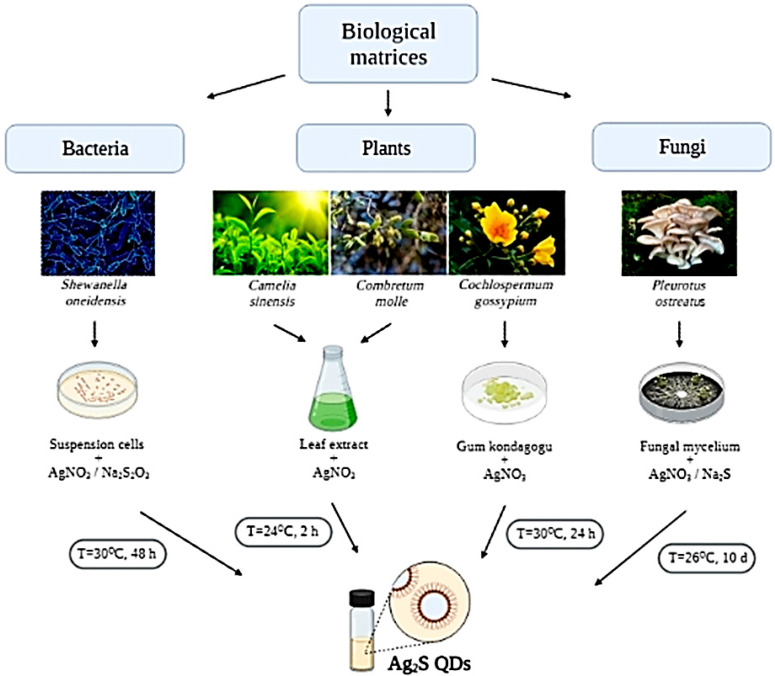
Synthesis of Ag_2_S QDs using different biological matrices.

Different living organisms interact differently with metal ions which form NPs of different chemical compositions. That is why the process of biological formation of QDs using the so-called “green” synthesis approach is still insufficiently studied [59]. NPs can be synthesized from various biological matrices using bacteria, fungi, plant cells, etc. The synthesis, in turn, is divided into extracellular and intracellular. Figure 3 schematically illustrates the Ag_2_S formation process using various biological matrices.

In bacteria, for example, a key element in the intracellular synthesis of QDs is the cell wall. Metal ions interact with the negatively charged cell wall, while cell wall enzymes reduce metal ions to QDs. Extracellular synthesis of QDs mainly occurs with the act of the enzyme nitrate reductase.

Plant-mediated quantum dot synthesis is faster, and the resulting NPs are more stable compared to microbe-mediated synthesis. Important components of plant extracts are amino acids, terpenoids, flavonoids, phenolic, and other compounds. Phenols play the role of reducing agents that stabilize NPs due to their interaction with their carboxyl groups, ensuring the production of stable QDs. [60]. The mechanism of biosynthesis of QDs depends on the selected biological object, the solution of the appropriate inorganic salt, the pH level, and NPs location.

The purpose of biological synthesis is primarily not to use toxic reducing agents, high temperature, and pressure conditions. There are certain restrictions on the use of NPs in biology and medicine. These eco-friendly gentle approaches are discussed below. “Green” synthesis methods and basic optical features of Ag-based QDs are presented in Table 3.

## 5. Ag_2_S QDs Biosynthesis

Bacteria *Shewanella* are often used for the synthesis of Ag_2_S QDs, which is reported, in particular, in Reference [61]. The size of Ag_2_S NPs, obtained using nontoxic reagents in a water solution at an ambient temperature on air, was 9–12 nm. The effect of certain parameters, such as temperature, reagent concentration, incubation time of bacterial strains on the QY, and size of Ag_2_S NPs, produced by bacteria *Shewanella oneidensis* in an aqueous solution with the addition of silver nitrate and sodium thiosulfate was also investigated. The optimal temperature range for the large-scale production of *S. oneidensis* biomass is 25–30 °C. The same temperature is suitable for the bacteria-mediated reduction of metal ions. The QY of NPs depends on the incubation time of the bacterial culture and temperature range. The particle sizes were 7–9 nm.

Ag_2_S QDs can also be synthesized using bacteria *Bacillus subtilis,* as reported in Reference [68]. The authors have established that flagellin is the main protein, providing the synthesis process [63]. The authors also note the key role of methyl lysine in the formation of silver sulfide QDs. The indicated study allows us to conclude the prospects for using *B. subtilis* for the production of Ag_2_S since the obtained NPs were homogeneous, their size did not exceed 10 nm, and they demonstrated high chemical stability.

Our research group has recently successfully synthesized Ag_2_S using the fungus matrix. As initial components for biosynthesis, we used AgNO_3_ and Na_2_S. The absorption maxima of the produced quantum dots were 315 and 470 nm. Luminescent maxima corresponded to 520 nm. The size of synthesized NPs was in the range of 10–15 nm. All experimental details are presented in Reference [14].

It was found that Ag_2_S QDs possessed an antibacterial effect, and they did not have significant genotoxic effects. It has been shown that the fungus can stabilize produced NPs of Ag_2_S, and due to the high luminescent intensity and nanosize, these QDs can penetrate the cell membrane and serve as intracellular luminescent probes. Organic molecules used as matrices (chitosan, STAB (sodium triacetoxyborohydride), starch) shift luminescence to the ultraviolet region, while chemically synthesized Ag_2_S QDs are characterized by NIR luminescence [14].

Another study was devoted to obtaining Ag_2_S QDs with the aid of chitosan, *Camellia sinensis*, *Combretum molle,* or *Acacia mearnsii* extracts. The authors reported that AgNO_3_ and thiourea were used as silver and sulfur initial components. QDs were obtained by surface modification with biocompatible compounds. The authors studied the absorption spectra of Ag_2_S NPs obtained with *C. sinensis*, *C. molle*, *A. mearnsii,* and chitosan under different pH conditions. For *C. sinensis,* a peak at 387 nm was shown at basic pH, while 402 nm was observed at acidic pH. For chitosan, the peak in an alkaline medium was shown at 343 nm, while in an acidic medium—350 nm. For *C. molle*, the peak at 360 nm was observed at basic pH, whereas the maximum at 365 nm corresponded to acidic pH. For *A. mearnsii*, the maximum absorption at 352 nm was shown in an alkaline medium and the maximum at 354 nm in an acidic medium. Eventually, the authors have concluded that basic pH level was the optimum environment for the production of Ag_2_S with the mentioned plant material. The diameter and morphology of the synthesized Ag_2_S QDs were studied with TEM. *C. sinensis*, *C. molle*, *Acacia mearnsii,* and chitosan produced mostly spherical NPs. It is important to note that Ag_2_S QDs were smaller in size at higher pH than the NPs synthesized at a lower pH. Ag_2_S NPs that were stabilized by chitosan had the smallest diameter, probably due to a large number of free amine groups in the chitosan structure. Overall, the above findings allow us to suggest that the diameter and chemical stability of the synthesized Ag_2_S QDs strongly depended on the pH level in the medium [63].

Another research presents a biosynthesis of Ag_2_S QDs using a plant of the *Cochlospermum genus*. In the natural environment, this plant grows in the woods of India. The resin of this tree is associated with substituted rhamnogalacturonans. It was found that Ag_2_S QDs had a wide maximum from 400 to 800 nm since they had a narrow band gap. The authors concluded that Ag_2_S QDs had a high photocatalytic activity at ultraviolet and visible light since they had a higher absorption at the ultraviolet and visible range. The microanalysis of the elements and an average particle diameter of Ag_2_S QDs were performed by the XRD. Most of the particles had an elongated dumbbell shape with a length of 100–550 nm and an average particle diameter from 45 to 54 nm. The produced QDs had a PL maximum at 470 nm and maximum excitation at 350 nm. Thus, *Cochlospermum* is an effective biomatrix for the “green” synthesis of Ag-based QDs. It determines the morphology and diameter of the synthesized Ag_2_S [68].

In another study reviewed, a cellulose/Fe_3_O_4_ nanosystem was used for the growth of Ag_2_S QDs crystals with the aid of “green” synthesis using leaves and seeds extract of *Pistacia atlantica*. The obtained Ag_2_S QDs were applied to neutralize organic dyes within a short period. The plant extract compounds were critical agents during the synthesis and stabilization of Ag_2_S QDs [69]. Biopolymers-based nanocomplexes demonstrate remarkable biocompatibility and biodegradability while improving structural and functional features due to their biological or inorganic compounds. Cellulose templates including heteroatoms such as nitrogen or sulfur have a high potential for their biotechnological application. They can assemble metallic NPs on polymer surfaces, thereby improving their stability. The authors have established that the size of Ag_2_S QDs obtained with leaves extract ranged from 12 to 15 nm, whereas the size range of Ag_2_S QDs obtained with seeds extract was 8–12 nm. These NPs showed a well-observed lattice structure that confirms the high crystallinity of Ag_2_S in both samples [69].

A new study presented rosemary leaves (*Rosmarinus officinalis*) water extract used for biosynthesis of Ag_2_S nanocrystals at ambient conditions [70]. The initial components used were silver nitrate (AgNO_3_) mixed with the above-mentioned plant extract. The reaction medium became black and had an absorption maximum at 355 nm, which was inherent to Ag_2_S because of the surface plasmon resonance phenomenon. The Fourier-transform spectrum of infrared spectroscopy of *R. officinalis* extract had a complex of peaks, reflecting its complicated origin. A wide absorption band at 3437 cm^−1^ is inherent to the alcohol/phenol–OH valence fluctuations, carboxylic–OH stretching, and N-H fluctuations of amides. TEM analysis confirmed the spherical morphology of such QDs and their diameter from 5 to 40 nm; an average size was 14 nm. The obtained green-synthesized Ag_2_S QDs were tested for their antimicrobial efficiency against *Escherichia coli*, *Staphylococcus aureus*, *Shigella,* and *Listeria* strains. It was found that Ag_2_S QDs revealed their bactericidal properties at different concentrations. Eventually, the authors concluded that the leaves extract of *R. officinalis* was an effective plant matrix for forming Ag_2_S QDs, which was confirmed by various physical parameters. Moreover, it is important to note that these NPs have a high bactericidal effect [70].

It was shown that Ag_2_S QDs could also be synthesized in a starch matrix. Optical, structural-morphological, and thermal techniques investigated the obtained QDs. XRD spectra indicated the availability of nanostructured silver at the cubic phase and silver sulfide at the monoclinic phase in the test samples. A commercial probe of farina matrix had 27% amylose, 12.5% natural moisture, and an average particle diameter of 32 µm. A TEM investigation revealed some agglomerates in the samples, but most QDs were homogenous in the initial starch matrix. The particle diameter was around 8 nm. UV-Vis spectra of produced Ag_2_S QDs revealed the first peak pointed at 380 nm, shifting to the higher wavelengths, while the second peak was at around 420 nm, shifting to blue when the solution concentration decreased. Subsequent dilution led to the appearance of an acute peak at 398 nm, and its position did not depend on the concentration of an initial reaction medium [64].

Another study described the synthesis of Ag_2_S nanorods by conjugating them with the bovine serum albumin (BSA) at ambient conditions. It is important to note that biofunctionalization with antibodies, peptides, or proteins is biocompatible and more preferable for biomedical purposes. It can modify nanocrystal surface via interaction of functional groups (electrostatic bindings) with subsequent application in optical devices, drug delivery, biosensing, and other fields. Binding protein molecules achieve steric stabilization of nanoscale particles to the nanoparticle surface. Amino acid residues that are part of the BSA structure can form binding centers to protein molecules on the nanosurface, which is a prerequisite for creating bioconjugates. The synthesis of Ag_2_S nanorods was carried out as a two-stage process. The first stage included obtaining the Ag-BSA complex by mixing AgNO_3_ with BSA components. The second stage included producing Ag_2_S nanorods by adding thioacetic acid into the reaction mixture at room temperature. It was found via TEM that synthesized nanorods were homogenous. The rims of the nanosystems were illegible and amorphous, which was caused by the presence of BSA residues. Presumably, the nanorods were capped by BSA. Ag_2_S nanorods were established to have a strong crystalline structure and could be attributed to monoclinic α-Ag_2_S. The particle diameter was 40 nm on average and 220 nm lengthwise.

The luminescence spectrum of prepared nanosystems was measured at the excitation of 378 nm. It revealed a symmetric peak at 474 nm. It is of interest that the authors studied the impact of BSA concentration on the synthesis of these nanorods. The BSA concentration of approximately 1–2 mg/mL was optimal for the growth and formation of nanosystems. At a low BSA concentration of about 0.5 mg/mL, mostly spherical Ag_2_S NPs were formed. In contrast, high BSA content of 4 mg/mL did not cause any significant alteration to the nanorods structure [71].

## 6. Ag_2_Se QDs Biosynthesis

Silver selenide (Ag_2_Se) quantum dots are appealing to study since they express specific properties in many applications: refrigerants, energy storage devices, thermoelectric electrodes, optoelectronic equipment, photo filters, sensitive probes, solar cells, etc. The successful synthesis of Ag_2_Se QDs by various natural capping compounds, namely *C. sinensis*, ascorbic acid, chitosan, and glucose, has been reported [72]. Spectrophotometric studies of Ag_2_Se QDs were also performed, which were characterized by a peak at 360 nm (glucose, ascorbic acid), 359 nm (chitosan), and 361 nm (*C. sinensis* extract). The results of absorption spectrophotometry measured for glucose, *C. sinensis,* and chitosan conformed with TEM investigations, which demonstrated that an average diameter of synthesized Ag_2_Se was 8–30 nm. On the other hand, ascorbic acid-capped quantum dots were 96 nm in diameter. Absorption maxima were blue-shifted towards the band gap value of Ag_2_Se, which meant the production of Ag_2_Se QDs. Luminescent properties of these Ag_2_Se QDs were also explored under different excitation bands: 240–550 nm. The synthesized Ag_2_Se QDs had various morphologies, namely spheres, sheets, nanorods, and nanocubes, with a diameter range from 8 to 96 nm that directly depended on the selected initial biomatrix.

In another study, the authors explored the effect of stabilizing agents on the size and shape of the produced QDs. TEM analysis revealed that the QDs had different morphologies: rods, spherical, or cubes with an average diameter from 8 to 96 nm depending on the capping organic molecules. It was observed that spherical and rod morphology was inherent to *C. sinensis*-based Ag_2_Se QDs with the size of 30 nm; QDs synthesized by glucose had clearly spherical and cubic morphology with similar size of 31 nm; QDs produced by ascorbic acid had a spherical morphology and a diameter of about 96 nm, and QDs synthesized by chitosan had a strong spherical and rod structure with a particle diameter of 8 nm. An orthorhombic phase was shown for these nanocrystals by X-ray diffraction [65].

Ag_2_Se NPs capped by starch matrix have also been synthesized at room temperature using NaBH_4_ as a reductant. pH influence and initial components concentration were studied for their effect on the diameter and morphology of QDs [73]. Starch was applied as an initial matrix due to its safety, ecological friendliness, biocompatibility. Moreover, it can be applied to biomedical research. The acidity of the reaction medium is a significant factor affecting the diameter of the QDs. This can be explained by the fact that pH affects the distribution of functional -OH groups of starch. Lowering the pH level changes the chemical structure of starch. It should be noted that a higher pH level increases the percentage of functional -OH groups of starch, which causes the formation of smaller-sized QDs. The other issue investigated was the morphology of quantum dots. The synthesized Ag_2_Se QDs were not adhesive. The authors determined that the main factor influencing the diameter and morphology of QDs is the concentration of precursors. The use of higher concentrations of the precursors increased the period of the NPs growth that caused the appearance of various shapes of NPs [74].

For the first time, synthesis of Ag_2_Se QDs using the one-pot ultrasound irradiation method by mixing water solutions of AgNO_3_ and H_2_SeO_3,_ that act as Ag and Se precursors, with D-fructose capping has been reported [75]. The absorption maximum of Ag_2_Se was identified at 413 nm. The obtained NPs were heterogeneous with a diameter of 5 to 40 nm. Silver selenide QDs demonstrated a concentration-dependent bactericidal effect *against E. coli*, *S. aureus*, *P. aeruginosa,* and *P. typhimurium*, and a cytotoxic effect against human fibroblast cell line. This study confirms the effectiveness of using organic molecules that can provide the synthesis of Ag_2_Se by ultrasound irradiation using a one-pot, inexpensive, and not a time-consuming method, which allows producing highly stable luminescent nanomaterials for biotechnological research.

In a recent study, authors have developed a novel approach for the “green” synthesis of silver selenide QDs with *Saccharomyces cerevisiae* culture. TEM analysis confirmed that the obtained Ag_2_Se quantum dots were homogeneous and had a diameter of about 4 nm. Due to the use of *S. cerevisiae* the fluorescence intensity increased four times. Ag_2_Se QDs proved to be biocompatible, nontoxic, with a high luminescence intensity, and opening promising opportunities for bioimaging [75].

## 7. Ag_2_Te QDs Biosynthesis

As far as we know, several research papers have been published to date on obtaining Ag_2_Te nanotubes, nanorods, nanocubes [34,76]. However, little is known about attempts to synthesize luminescent Ag_2_Te QDs with NIR luminescence. For conversion Ag_2_Te to the hydrophilic phase, the surface of these nanocrystals needs to be modified by ligand exchange. It is particularly important to consider the hydrodynamic dimensions of QDs for intracellular visualization because too large size limits the diffusion of QDs within the cytoplasm. Therefore, several methods for modifying the surface of Ag_2_Te QDs while preserving their photostability and brightness have been proposed in the published reports. In the study [77], the authors showed that the decrease of PMAC concentration was accompanied by a luminescence shift of Ag_2_Te to the NIR-II wavelength range. It was also shown that thiol ligands could chelate metal ions, forming high-affinity metal-ligand complexes.

## 8. AgInS_2_ QDs Biosynthesis

CuInS_2_, CuInSe_2,_ or AgInS_2_ are intensively studied ternary QDs I-III-VI due to their low toxicity compared to common semiconductor QDs II-VI that are usually formed by heavy metals core with pronounced toxicity [48]. Fluorescent QDs of this structure can be used to develop a fluorescent sensor to detect microconcentrations of substances, organic molecules, microorganisms, etc. For instance, the study proposed identifying ascorbic acid molecules [66] by developing a “green” chemistry approach for the production of AgInS_2_ QDs. Produced in this way, AgInS_2_ QDs had strong NIR luminescence, and the luminescent peak corresponded to 680 nm. QY was also determined to be 10.3%. Particle diameter was calculated according to TEM measurements as 2.5 nm. Their crystalline structure was confirmed by XRD. In this investigation, the connection between the concentration of ascorbic acid and the luminescence intensity of the synthesized AgInS_2_ nanocomposites was studied. It was found that the luminescence intensity of AgInS_2_ increased with an increment of ascorbic acid concentration. The authors explained this result as a coating of the surface defect on AgInS_2_ with ascorbic acid. They also concluded that AgInS_2_ QDs were effective nanomaterials for creating high-precision sensors for biomedical research.

Another study reported on the production of AgInS_2_/ZnS nanocomposites through biological synthesis in a water solution using AgNO_3_, InCl_3_ sodium citrate, and TGA as a stabilizer. The luminescence intensity of the prepared AgInS_2_ core doubled after the formation of the ZnS shell. Tetragonal lattice was inherent to these QDs. HRTEM analysis confirmed that these nanocomposites had a spherical morphology predominantly. Cytotoxic studies were also performed on HeLa cells after exposure to different amounts of AgInS_2_/ZnS. The obtained nanocrystals were found to be nontoxic even during a long incubation period. AgInS_2_/ZnS demonstrated high compatibility with all experimental cell lines. The percentage of viable, intact cells remained at over 75%, an important parameter for biological utilization [67].

A prerequisite for obtaining ternary Ag-containing QDs is the availability of silver nitrate and the corresponding inorganic components (sulfur, selenium, InCl3) in addition to the selected biomatrix. Enzymes, alkaloids, flavonoids, organic acids can be used as stabilizers during the biosynthesis process.

## 9. Advantages of “Green” Synthesis of Ag-Based QDs

“Green” synthesis of nanomaterials is a promising technology compared to the commonly used chemical or physical approaches. Biological matrices are so-called “nanofactories” capable of producing NPs of different morphology and chemical composition. For instance, plant extracts and fungi are widely used since they are available throughout the year, are inexpensive, nontoxic, and contain a large number of secondary metabolites, tannins, proteins, organic acids, which can act as capping compounds that ensure the efficiency of “green” synthesis of semiconductor QDs [12].

There are twelve points of “green” chemistry, followed by researchers around the world, including the principle of “green” synthesis of nanomaterials: nuclear economy, prevention, less hazardous chemical synthesis, safer solvents and auxiliaries, development of safer chemicals, design for energy efficiency, reduction derivatives, use of renewable raw materials, catalysis, real-time analysis for pollution prevention, design for degradation, essentially safer chemistry for accident prevention. Three main stages are determined in the process of biosynthesis: the selection of biological organisms as the starting matrix, the selection of initial reagents, and the selection of appropriate reaction conditions [78]. The main advantages of “green” synthesis are method reproducibility, low cost and nontoxic materials, ambient conditions, and availability of raw materials thought the year.

As suggested above, the three most common sources for “green” QDs synthesis are bacteria, fungi, plants, various biomolecules, each of which has its own advantages. Bacteria are characterized by resistance to the metal salts in the solution in two ways: intracellular chelating of metal ions or outflowing them outside. The fact that bacteria can accumulate and convert inorganic ions was established about 30 years ago. Engineers and scientists now explore such unique properties of microorganisms to create different types of nanocomposites, alloys, metal oxides, or some nanosized compounds. *Enterobacteriaceae*, *Bacillaceae*, *Shewanellaceae*, *Pseudomonadaceae* are the most widespread microbial families for producing different types of QDs and NPs (Au, Ag-based, Pt, Pd, CdS, CdSe, etc.). The efficiency of biosynthesis is determined by the redox potential of a certain bacterium strain and bacterial resistance to metal compounds [79].

Fungi are suitable for large-scale cultivation in fermenters, producing saturated biomass. They can grow even on an inorganic substrate during the cultivation process, catalyzing a more efficient metal distribution. Fungi are producers of various enzymes, such as reductases, that provide biosynthetic pathways for nanomaterials creation [80]. The synthesis of NPs with the help of plant extracts significantly reduces the burden on the environment; it is safe and economically justified. Plant biomass is an easily accessible and safe natural material for biosynthetic developments. In addition to plant extracts, in vitro cultures can also be used for QDs biosynthesis. Several works indicate the possibility of producing semiconductor QDs through hairy roots culture [12,81,82,83].

A significant advantage of hairy roots compared to de-differentiated plant cells is their genotypic and phenotypic stability. They are easier to cultivate in vitro than callus or suspension cultures since growth regulators are not required. Hairy root culture allows you to estimate the accumulation capacity of the metal and the tolerance of roots to heavy metals. Although these cultures offer some advantages for studying metal uptake and tolerance in plants, they have some limitations associated with hairy roots in vitro. These limitations include the composition of the growth medium and bacterial contamination during aseptic culturing. Nevertheless, they can be successfully applied as in vitro production facilities to synthesize metal QDs [12,81,82,83,84], including Ag-based QDs.

## 10. Toxicity of Ag-Based QDs

A major drawback associated with the biomedical application of QDs is their potential toxicity caused by their chemical composition (heavy metal ions) or nano dimensional properties. Over the last two decades, reinforced attempts have been devoted to the development of cadmium-free QDs. So, silver-based QDs are used as a promising generation of QDs that are relatively nontoxic composites with a special interest in various biocompatible applications [85]. Hocaoglu et al. [86] have highlighted (DMSA)-coated Ag_2_S QDs as one of the most intensive luminescent, anionic, NIR-emitting QDs and shown that these particles were accumulated into HeLa cells and provided strong intracellular optical signals, quenching autofluorescence. The authors did not observe the toxic effects of 200 µg/mL QDs on HeLa cells but, at the same time, a 20% decrease in the viability of mouse embryonic fibroblasts (NIH/3T3) was observed in 24 h [86]. Another report confirmed that Ag_2_S QDs capped with DMSA had high biocompatibility and low toxicity due to the insoluble Ag_2_S semiconductor core. The results clearly demonstrated that DMSA-capped Ag_2_S QDs had neither cytotoxic nor genotoxic impacts in a cell line (V79) in medically suitable concentration range, but in high doses could only induce apoptosis, mediated by signaling pathways, including p53, survivin, or caspase [85].

Fructose was used as a natural raw component to synthesize Ag_2_Se and further evaluate their cytotoxic and antimicrobial effects [87]. The biological activity of silver selenide was assessed using standard cytotoxic and bactericidal tests: MTT assay and disc diffusion method. Human fibroblasts (ATCC) were incubated with different concentrations of Ag_2_Se QDs for 24, 48, and 72 h. The obtained results indicated manifestation of cytotoxicity reactions compared to the control ones. The «green» synthesized Ag_2_Se caused a significant decrease in the mitochondrial activity of ATCC cells in comparison to the untreated cells. On the other hand, the study of antimicrobial activity of Ag_2_Se QDs against *Escherichia coli*, *Staphylococcus aureus*, *Salmonella typhimurium,* and *Pseudomonas aeruginosa* showed no effect on the bacterial cell wall type. However, the differences in the toxic effect of Ag_2_Se on Gram-negative and Gram-positive strains occurred—silver QDs were toxic towards Gram (−) *E. coli*, mildly toxic against *S. typhimurium,* and had the lowest toxic influence on *P. aeruginosa*. But the studied Ag QDs demonstrated very high toxicity against the Gram (+) *S. aureus*. Moreover, increasing the Ag_2_Se QDs concentration expands the inhibition zone for all tested pathogenic bacteria. In general, Ag_2_Se QDs, which were synthesized through a one-pot «green» approach with low toxicity, seem to be promising candidates for biomedical application [87].

Ag_2_Te is the least analyzed member of the silver chalcogenide family, and there is only scarce information concerning the cytotoxicity of these QDs. Their biological applications require sophisticated surface functionalization approaches. It is known that the surface coupling of Ag_2_Te QDs with a modified polyethylene glycol method was developed that can provide water solubility and biocompatibility of functionalized silver telluride [30]. The cytotoxicity of Ag_2_Te QDs was assessed on mouse fibroblasts cells (L929) for 24 and 48 h incubation at a concentration of 1 mg/mL. The test indicated a minor impact on the cell viability of Ag_2_Te QDs (more than 90% of cells remained viable after 48 h exposure with these QDs). To evaluate the potential apoptotic and necrotic effects of Ag_2_Te on the tested cells, the latter were stained with anti-annexin and propidium iodide dyes, and no significant increase of apoptosis and necrosis was established compared to the untreated cells. Moreover, L929 cells treated with Ag_2_Te QDs in different concentrations demonstrated a low level of ROS release induced by silver telluride. Cytotoxicity assays of modified Ag_2_Te QDs showed minor toxic effects and good biocompatibility. Thus, Ag_2_Te QDs could be developed as innoxious NIR-II luminescent labels for bioimaging approaches in clinical research [30].

AgInS_2_ dichalcogenide can be successfully used for in vitro and in vivo studies. The toxicity of micelle-encapsulated AgInS_2_ was assessed on mice, including tissues section analysis. The cytotoxicity of AgInS_2_ was also evaluated on human pancreatic tumor cells (cell line Panc-1), which remained viable after 24 and 48 h of incubation [88]. It was shown that even at a high concentration of QDs (up to 500 µg/mL), cells remained alive, which indicates minor cytotoxic effects associated with these nanomaterials. The minuscule size, NIR emitting luminescence, and low toxicity make the AgInS_2_ QDs promising contrast agents for tumor detecting and visualization [88].

To sum up, silver-based QDs can be novel functional materials for biomedical applications since they are highly luminescent with insignificant geno- and cytotoxic influence in vivo. They are considered a safe tool for diagnostics, gene delivery, or cell imaging.

## 11. Bioimaging Applications of Ag-Based QDs

Bioimaging with the aid of semiconductor nanomaterials is a promising technique due to its unique fluorescent properties compared to widely used organic fluorophores. This allows you to create effective biomarkers, biosensors, delivery of antitumor drugs to target cells. Optoelectronic properties of QDs include high QY of luminescence, wide absorption spectrum, and high photostability [89]. Quantum dots have significant advantages over organic dyes. Narrow size distribution, emission from a single light makes them most suitable for multiplex imaging. According to [90], some important parameters allow comparison of QDs and traditional organic dyes (Table 4).

The composition of living tissues includes various biomolecules (DNA, collagen, elastin) that emit light in a wide range, which covers both UV and visible areas. NIR emission is preferable for imaging of deep living tissues since autofluorescence attenuates. Thus, the luminescence at the wavelengths 750–940 nm and 1100–1700 nm, which is inherent to silver-based QDs, has undeniable advantages in such biomedical applications as dynamic cellular imaging, detection of intracellular structures, in vivo imaging for tumor detection, etc. To sum up, the advantages of fluorescence imaging using nanoprobes are as follows: (1) non-ionizing radiation is used for excitation; (2) obtaining high-quality real-time images, and (3) availability of the experiment.

One of the examples of silver-based QDs bioimaging applications is a study in which targeted cell imaging was performed using intrinsic NIR-II fluorescence of the DHLA-Ag_2_S QDs to demonstrate the feasibility of using bright Ag_2_S QDs as effective NIR-II emissive probes. Two cell lines, the human breast cancer cell line (MDA-MB-468) and human glioblastoma cell line (U87 MG), were used since they comprised two cell types with different levels of expression of two membrane receptors, αvβ3 integrin and epidermal growth factor receptor (EGFR). For their specific targeting to αvβ3 integrin and EGFR cyclic arginine-glycine-aspartic acid (RGD), peptide and cetuxima b protein were chosen. Using the coupling chemistry, they were bound to carboxyl groups of DHLA ligands on Ag_2_S QD surfaces. Ag_2_S simply bound to the cell membrane receptors by specific recognition of the ligands conjugated to QD surfaces and, thus, truly reflected the level of membrane receptor expression [91].

In another study performed to examine the process of angiogenesis, Ag_2_S QDs were shown to have a long period of chemical stability and fluorescence in vivo bioimaging tests. Photoluminescence of Ag_2_S QDs in the blood was monitored in real-time, which also did not indicate toxicity. The QDs scans have also been used as mapping to determine tumor sites, magnitude, and extent. This way of tumor mapping can be performed without the need for a biopsy. In order to estimate the margin of the tumor, a test was carried out using silica QDs, modified with RGDY peptides for the cartographic observation of breast cancer, to monitor by positron emission tomography [89]. Furthermore, NIR QDs were synthesized to detect Cu^2+^ ions with high sensitivity and monitor changes in their concentration through in vitro and in vivo fluorescence imaging studies [92]. In vitro and in vivo assays for bioimaging are presented in Table 5.

Ag_2_S QDs possess photostability and brightness suitable for stable and intense bioimaging. Unlike commonly applied heavy metal-based (cadmium or lead) QDs, Ag_2_S QDs have been verified through in vivo and in vitro studies as having no significant toxicity. Moreover, Ag_2_S QDs show an anticancer activity themselves, based on the photothermal effect. Chemotherapeutic agents can be efficiently conjugated to the Ag_2_S QDs surface due to electrostatic interaction, high affinity, and covalent binding. Thus, Ag_2_S QDs can simultaneously act as both an imaging agent and a therapeutic agent in imaging-based diagnostics, which inspired the creation of Ag_2_S theragnostic nanomaterials [24]. Non-invasive biomedical imaging methods are of growing interest in medical and clinical research due to their prognostic and diagnostic value for various pathologies.

According to [96] for tumor detection, QDs were conjugated with antibodies to prostate-specific membrane antigen. The authors observed the accumulation and maintenance of this antigen-antibody complex at the cancer growth site, which is the basis of targeted therapy for metastasis of the human prostate tumor.

**Figure 4:**
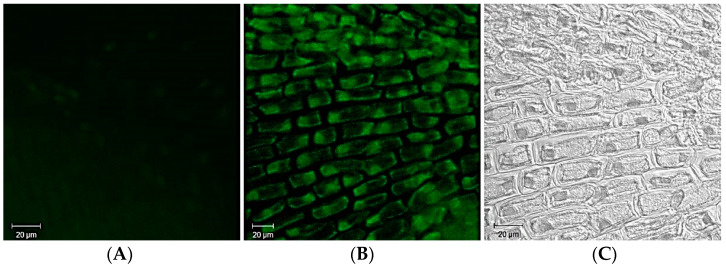
Confocal microscopy image of *Allium cepa* epidermal root cells. Exposure time 24 h: (**A**)—untreated cells; (**B**)—cells treated with Ag_2_S QDs; (**C**)—cells under transmitted light.

Fluorescent imaging in NIR-II, which ranges between 1000 and 1700 nm, is more suitable for diagnostic purposes due to the tissues’ reduced absorption and scattering. Due to their high photostability and negligible toxicity, Ag_2_S can be considered promising alternative candidates for NIR-II fluorescent imaging [97]. However, to the best of our knowledge, the use of Ag_2_S as luminescent probes in plant cells has not received due attention and coverage in the research literature yet. It is known that silver-based NPs can be used as a powerful tool for bioimaging because they have unique optical and plasmonic properties: high molar extinction coefficients, resonant Rayleigh scattering, intense luminescence. We have investigated the luminescent properties of silver sulfide QDs synthesized by the “green” method using fungi, their penetration, and localization in plant cells. The produced Ag_2_S QDs had a luminescence in the range of 520–550 nm, corresponding to the green visible light spectrum. The produced Ag_2_S quantum dots were 10–25 nm in diameter. It has been established that silver sulfide QDs penetrated *Allium cepa* root cells and localized mostly in the nucleus of epidermal and meristematic cells of the root tips cell (Figure 4).

Thus, we demonstrated efficient biosynthesis of extracellular highly stable, spherical silver sulfide QDs produced by the fungus matrix. Given their luminescent properties and lower environmental toxicity, they are promising luminescent probes for cell biology [14].

Ag_2_Se nanocrystals can also be used in various fields of optoelectronics and biotechnology. However, there is still insufficient information on using this NIR emitted nanomaterial for tissue imaging in vivo. The challenge is to obtain Ag_2_Se of small diameter due to the low solubility constant and rapid growth of nanocrystals. The luminescent maximum of Ag_2_Se QDs shifts to the mean values of 700–820 nm. A commonly used organic fluorophore indocyanine green was used as a control in bioimaging experiments with Ag_2_Se. Photofading of indocyanine green was observed after 120 min of irradiation, while Ag_2_Se QDs retained 65% of their photostability even after 300 min of continuous excitation [98,99]. For instance, Tang et al. [93] studied blood clearance, transformation distribution, and toxic effects of Ag_2_Se QDs functionalized by polyethylene glycol (PEG) after intravenous injection into mice. Ag_2_Se NPs mainly accumulated in the liver and spleen, excreted by the kidneys within one day; however, selenium accumulated in the body over time. Eventually, PEGylated Ag_2_Se QDs had a low toxic impact. These Ag-based QDs had significant potential for in vivo labeling studies. The produced Ag_2_Se QDs were characterized by NIR-II fluorescence in the wavelength 1000–1330 nm. Ag_2_Se QDs were photostable and water-soluble, which was an important parameter for their application in polychrome fluorescence in vivo [93].

Ag_2_Te QDs attracted attention in the fields of bioimaging, chemo/biodetection, and thermoelectric materials, but their biosafety has not been well assessed yet. The study [97] presented that the stability of Ag_2_Te QDs-PEG in the mice depended on the organ of their accumulation and the time. PEG-coated Ag_2_Te were stable in the spleen within 28 days but were destroyed in the liver within 7 days. Then PEG-coated Ag_2_Te were excreted from the organism. PEGylated Ag_2_Te QDs did not show significant negative effects on the body weights and organ indices, indicating that Ag_2_Te QDs have good biocompatibility in mice. This could be considered a prerequisite for their further biomedical applications [94].

The use of AgInS_2_ nanocrystals emitting in the near-infrared range is also suitable for tracking deep carcinoma. AI-III BVI semiconductors, such as AgInS_2_ QDs, attract research attention because they do not contain heavy metal ions (cadmium, lead, mercury), are low toxic, and emit NIR. Their band gap is within 1.87–1.98 eV, depending on the phase of the crystal. Thus, nanosized AgInS_2_ are potentially promising fluorescent probes for efficient in vitro and in vivo imaging and biosensor generation. The emission peaks of these QDs range at 800 nm. For instance, the water-soluble AgInS_2_ QDs have optical stability and can be applied for tumor detection in both in vitro and in vivo. The authors confirmed the suitability of AgInS_2_ nanocrystals as effective NIR luminescent probes in experiments where these QDs were administered intravenously to mice with deep tumors to obtain high-quality images of cancer localization [100].

The signals from AgInS_2_ QDs are pseudo-red, and the background fluorescence is shifted in the short-wave region. It was established that nanocrystals capped in micelles accumulated in tumors following 15 min after injection. AgInS_2_ QDs encapsulated in micelles are suitable for the drug delivery of cancer therapy and diagnostics since they can be used to distinguish the tumor area from the background tissue. Thus, anticancer drugs-capped AgInS_2_ QDs release target substances into carcinoma over a long period [88].

Researchers have focused on preparing water-soluble AgInS_2_/ZnS emitting as novel and safe probes for imaging in the NIR spectrum. It should be noted that triple I–III–VI QDs attract attention due to a prolonged time of luminescence in the infrared region. Owing to their optical parameters, they are suitable for bioimaging in vivo. In particular, PEGylated AgInS_2_/Zn_S_ QDs and fluorescein-conjugated tomato lectin were administered intravenously to mice 3 days after a breast tumor was irradiated. The labeled tomato lectin was used for imaging endothelial cells lining the tumor microvasculature, distribution of QDs in the tumor area was monitored by fluorescent microscopy [100].

In order to obtain organic dye-water soluble AgInS_2_/ZnS QDs coupled chromophores, two paths were described—the conjugation of polymer-encapsulated water-soluble QDs and carboxylic acid surface caps with amine functional organic dyes via PEG carbodiimide and the water-solubilizing by silane coating. Further in vivo imaging showed the bioimaging potential of PEGylated QDs and its distinctiveness in localization features in mice models [99]. The method of synthesis of ternary AgInS_2_, quaternary AgZnInS, AgInS_2_/ZnS, and AgZnInS/ZnS nanocomposites by cation exchange was shown by Song et al. in which low cytotoxicity was observed in vitro, so these NPs showed highly advantageous possibilities in clinical applications [95].

## 12. Conclusions

Silver-based QDs are essential elements in many electrochemical, optical and biochemical devices and processes, and are considered nontoxic nanomaterials for biosensors or imaging in living systems. Here, we summarize the data available on physicochemical peculiarities of biosynthesis of Ag-based QDs using different biological matrices such as microorganisms, fungi, plant extracts, and biomolecules. The advantages of “green” synthesis over chemical methods are also discussed. For bioimaging, NIR luminescence is preferable, since new QDs that emit within 700–900 nm are being developed. Infrared radiation penetrates most deeply into the tissues without damaging them. Therefore, Ag-based QDs are of particular interest. Future studies will include the development of QDs conjugates for cell tracking and diagnostics of various diseases, including cancer. These new imaging agents will also be useful for creating precise biosensors (including SERS-based nanoplatforms), drug delivery systems, long-term multicolor cell imaging, and other biomedical research.

## Figures and Tables

**Table 1:** Classification of Ag-based QDs.

Type of Silver-Based QDs	Band Gap (eV)	Crystal Structure	Phase Transition Temperature (°C)	References
Chalcogenides				
Ag_2_S	0.9–1.1	Monoclinic acanthite	Below 179	[23]
		Body-centered cubic argentite form	Above 180	
		Face-centered cubic	Above 586	
Ag_2_Se	0.02–0.22	Orthorhombic structure	~133	[29]
		Body-centered cubic form	Until 897	
Ag_2_Te	0.67	Monoclinic phase (β-form)	Transition at ~150	[32]
		Cubic phase (α-form superionic conductor)		
Ternary dichalcogenides	1.8	Cubic structure	>100	[40]
AgInS_2_				
AgInSe_2_	1.24–1.53	Chalcopyrite phase	300	[46]
		Metastable orthorhombic phase	250	
Quaternary dichalcogenides	1.7	Hexagonal structure	<100	[48]
ZnAgInS				
ZnAgInSe	1.2	Orthorhombic	200–250	[47]

**Table 2:** Methods of chemical synthesis of Ag-based quantum dots and their characterization.

Type of Quantum Dot	Chemical Synthesis Method	Average Diameter (nm)	Morphology	Photoluminescence (nm)	Crystal Lattice Structure	References
Ag_2_S	Single source precursor	5–10	Spherical	543	Orthorhombic or α-phase sulfur	[52]
	Sol-gel synthesis	30–60	Thin films	-	-	[21]
	Hydrothermal method	70–90 in length	Rice-shaped	-	Monoclinic	[51]
	Gamma-ray irradiation	200–500	Rod-like	-	Monoclinic	[50]
	Hydrothermal method	1.45–5.20	Spherical	748–840	Monoclinic	[22]
	Pyrolysis method	10.2 ± 0.4	-	1058	-	[21]
	Hot-injection method	1.5–4.6	Spherical	690–1227		
	Hydrothermal method	2.6–3.7	Spherical	687–1096		
	Microwave-assisted synthesis	5.7 ± 0.93	-	1062		
Ag_2_Se	Co-precipitation method	5–30	Wire-type	700–1330	Orthorhombic	[28]
	Solvothermal method	3.4	Spherical		ẞ-Ag_2_Se	
	Hydrothermal method	60–80 in length	Rice-shaped		-	
	Hydrothermal method	3.1–3.9	Spherical	1080–1330	Orthorhombic	[28]
	Hydrothermal method	2	Spherical	-	Orthorhombic	[25]
Ag_2_Te	Hydrothermal method	200	Wire-type	995–1300	Irregular dendrites	[56]
	Solvothermal methods	10	Spherical	-	Monoclinic	[31]
	One-pot aqueous Synthesis	3.8–4.7	-	995–1068	Monoclinic	[30]
	Hydrothermal method	2.4 ± 0.9	Spherical	1320	Monoclinic	[37]
AgInS_2_	Hot-injection method	3.7–4.3	Spherical	-	-	[39]
	Microwave synthesis	20–80	-	520–650	Tetragonal	
ZnAgInSe	Synthesized in Aqueous phase	3.5–4	Spherical	450–700	Orthorhombic	[47]
	Hydrothermal synthesis	1.5–4.5	Spherical	450–700	Cubic	[47]

**Table 3:** «Green» synthesis, photoluminescence, and morphology of Ag-based quantum dots.

Type of Quantum Dot	Living Organism/Derivatives/Biomolecules	Average Diameter (nm)	Morphology	Photoluminescence (nm)	Crystal Lattice Structure	References
Ag_2_S	*Shewanella oneidensis* MR-1	6–12	Spherical	-	Monoclinic	[61]
	*Camellia sinensis*	~20	Spherical	387–402	Monoclinic	[62]
	*Comtretum molle*			360–365		
	*Acacia mearnsii*			352–354		
	Chitosan			343–350		
	*Cochlospermum gossypium*	48–54	Spherical and cubic	500	Cubic and individual spherical particles	[63]
	*Pleurotus ostreatus* (Jacq.) P. Kumm. (strain 551)	10–17	Spherical	520	-	[14]
	Sago starch	9.5 ± 3.6	Spherical	-	Monoclinic	[64]
Ag_2_Se	Green tea	30	Spherical and rod	240–330 390–550	Orthorhombic	[65]
	Glucose	31	Spherical and cubic			
	Ascorbic acid	96	Spherical			
	Chitosan	8	Spherical			
	Glucose	2.4 ± 0.5	-	561–705	Orthorhombic	[27]
AgInS_2_ AgInS_2_/ZnS	Shell precursors (ZnAc_2_ and thiourea)	3.2–3.4	Spherical	667–677	Tetragonal	[66,67]

**Table 4:** Optical parameters of organic dyes and QDs.

Parameter	Dye	QDs
Absorption spectrum	Narrow	Broad and gradually increasing towards shorter wavelength
Emission spectrum	Broad	Narrow, symmetrical
Quantum yield (QY)	High-quality dyes and QDs have similar QYs	
Fluorescence lifetime	5–20 nanoseconds	50–200 nanoseconds
Photostability	Poor, rapid photobleaching	Highly stable

**Table 5:** In vitro and in vivo assays for bioimaging of Ag-based QDs.

Type of QDs	Cell Line/Organism	Fluorescence (nm)	Route of Administration	Reference
Ag_2_S	Mouse fibroblast L929 cell line	1100–1700	Cells were fixed in 4% paraformaldehyde and treated with QDs (in vitro studies)	[91]
	Human malignant glioma U87 MG cell line			
	Human breast cancer MDA-MB-468 cell line (ATCC)			
Ag_2_Se	Male CD-1 (ICR) mice	700–820	Intravenous injection (in vivo studies)	[93]
Ag_2_Te	Male ICR mice	900–1300	Intravenous injection (in vivo studies)	[94]
AgInS_2_	Radiation induced fibrosarcoma (RIF) cells	800	Intravenous injection (in vivo studies)	[88]
	Human peripheral blood monocyte-derived macrophages (MDM)			
AgInS_2_/ZnS	Human hepatoma cell line (Hep G2)	500–700	QDs delivered into Hep G_2_ cells and specifically combined with antigens (in vitro studies)	[95]
